# Poly(3-Hydroxybutyrate-*co*-3-Hydroxyvalerate): Enhancement Strategies for Advanced Applications

**DOI:** 10.3390/polym10070732

**Published:** 2018-07-03

**Authors:** Ariagna L. Rivera-Briso, Ángel Serrano-Aroca

**Affiliations:** 1 Escuela de Doctorado, Universidad Católica de Valencia San Vicente Mártir, C/Guillem de Castro 65, 46008 Valencia, Spain; aribri@mail.ucv.es; 2 Facultad de Veterinaria y Ciencias Experimentales, Universidad Católica de Valencia San Vicente Mártir, C/Guillem de Castro 94, 46001 Valencia, Spain

**Keywords:** poly(3-hydroxybutyrate-*co*-3-hydroxyvalerate), mechanical strength, thermal properties, electrical properties, wettability, biological properties, antimicrobial activity, water sorption, scaffolds, advanced applications

## Abstract

Poly(3-hydroxybutyrate-*co*-3-hydroxyvalerate), PHBV, is a microbial biopolymer with excellent biocompatible and biodegradable properties that make it a potential candidate for substituting petroleum-derived polymers. However, it lacks mechanical strength, water sorption and diffusion, electrical and/or thermal properties, antimicrobial activity, wettability, biological properties, and porosity, among others, limiting its application. For this reason, many researchers around the world are currently working on how to overcome the drawbacks of this promising material. This review summarises the main advances achieved in this field so far, addressing most of the chemical and physical strategies to modify PHBV and placing particular emphasis on the combination of PHBV with other materials from a variety of different structures and properties, such as other polymers, natural fibres, carbon nanomaterials, nanocellulose, nanoclays, and nanometals, producing a wide range of composite biomaterials with increased potential applications. Finally, the most important methods to fabricate porous PHBV scaffolds for tissue engineering applications are presented. Even though great advances have been achieved so far, much research needs to be conducted still, in order to find new alternative enhancement strategies able to produce advanced PHBV-based materials able to overcome many of these challenges.

## 1. Introduction

The current extent of technological development, prolonged life expectancy worldwide, and the associated challenges for patients of advanced age, are some of the factors that determine the growing demand for biodegradable materials. Thus, currently, there are a number of diverse medical treatments that depend on the use of biodegradable materials [1,2]. Additionally, there is a critical need to discover sustainable and affordable solutions in the face of the environmental devastation caused by plastic waste contamination [3] from the burning of fossil fuels, as well as the ensuing challenges of managing that waste in land and ocean [4].

As a result, there is a growing number of research efforts devoted to improving the properties of already existing biodegradable materials, such as polyhydroxyalkanoates (PHAs) [5].

**Figure 1:**
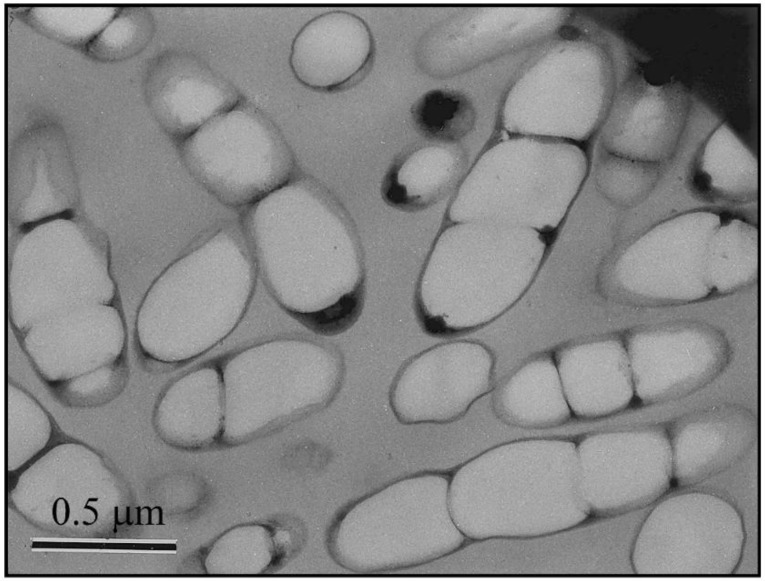
Transmission electron micrograph of thin sections of recombinant *Ralstonia eutropha* cells with polyhydroxyalkanoate (PHA) incrustations. Reproduced with permission from Ref. [9].

Among the most-produced and commercialised biopolymers in the world, PHAs stand out as a sustainable alternative, since they can be transformed into water and carbon dioxide in the presence of oxygen, or into methane under anaerobic conditions, by microorganisms present in water and soil [6]. The polyhydroxyalkanoates are a family of lineal biopolyesters composed of hydroxyalkanoate (HA) units, organised in a basic structure that is obtained through bacterial fermentation, and are presented as opening doors for a sustainable future [7]. These accumulate into polymers that are liquid, insoluble in water, mobile, and amorphous, taking the form of granules surrounded by a single layer of phospholipids, and containing polymerase and depolymerase enzymes [8,9]. These biopolymers are packed as granular inclusions in the cytoplasm of a wide variety of both Gram-positive and Gram-negative microorganisms when they are under conditions of nutritional deficiency in elements, such as nitrogen, phosphorus, magnesium, and sulphur, and in the presence of excess carbon [10]. These PHA inclusions are observed under the transmission electron microscope as spherical granules of varying sizes when thin sections of PHA-containing bacteria are observed (see Figure 1) [9].

The intracellular granules function as an energy storehouse for the cell that can convert them into carbon material when the external carbon source is depleted, or if the limiting nutrient is supplied again. The use of such a polymer is considered to be a strategy developed by bacteria to increase their chance of survival throughout changing environments [11].

The advantages that PHAs have over petroleum-based plastics, which make them greatly significant as the best candidates to replace conventional plastics, are that they can be synthesised from renewable carbon sources, and are biodegradable and biocompatible [12]. The most widespread and extensively studied member of this family is polyhydroxybutyrate or poly(3-hydroxybutyrate) (PHB), discovered in 1923 by Maurice Lemoigne of the Institut Pasteur [13]. PHB is a thermoplastic polymer with physical properties similar to some polymers derived from petroleum, such as polypropylene. It is highly biodegradable, non-toxic, and biocompatible. Among its most prominent characteristics are its high degree of crystallinity, its insolubility in water, and that it is relatively resistance to hydrolytic degradation [9].

It is well known that when PHAs are produced by Gram-negative bacteria, they can contain high levels of endotoxins, which can cause inflammatory, pyrogenic, and other reactions, which can limit their application as biomaterial if this compound is not removed during purification [14,15]. Thus, PHB with a low endotoxin level, suitable for biomedical applications, could be produced by chloroform extraction, and a much lower endotoxin level can be achieved from recombinant *Escherichia coli* by simple NaOH digestion [16]. This biopolymer can be used in agriculture, packaging for food products and medicine, although its application has been limited due to its poor mechanical properties, mainly on account of its high fragility [17,18,19].

A strategy widely used to improve the properties of PHB is the incorporation of various secondary monomers in the polymer chain to form copolymers. Thus, more than 150 different monomers can be combined to develop materials with drastically varying properties: copolymers of 3-hydroxybutyrate, 3-hydroxyhexanoate (PHBHHx), and poly(3-hydroxyoctanoate) (PHO) [20]. From all these bioplastics, one of the more promising materials for biomedical applications, is the poly(3-hydroxybutyrate-*co*-3-hydroxyvalerate) biopolymer, or PHBV, due to its null toxicity, high biocompatibility with many diverse types of cells, and that it nowadays can be produced at large scale [21,22,23]. Thus, haloarchaea belonging to Archaea domain, can accumulate PHBV with variable 3HV content and the produced PHBV contains low endotoxin levels, and exhibits excellent biocompatibility [24,25,26,27,28]. However, the PHBV produced by Gram-negative bacteria must be purified to remove endotoxins for biomedical applications. This removal during purification can be performed using oxidising agents, such as hydrogen peroxide [29].

The biocompatibility and biodegradability characteristics of PHBV make it an outstanding material with broad applications in a wide variety of sectors. The excellent properties of PHBV, such as its absorption capacity, biological origin, low cytotoxicity, piezoelectricity, and thermoplasticity, render it very promising for biomaterial applications, such as the fabrication of cardiovascular stents [30], drug release and transport systems [31,32], absorbable surgical sutures, and medical packaging [33]. In the field of tissue engineering, applications include the elaboration of tissue patches, biodegradable implants, biosensors, and the fabrication of porous scaffolds that allow the treatment of bone defects caused by diseases or injuries where conventional treatments are ineffective [34,35,36,37].

In other industry sectors outside of the biomedical field, there is a wide range of applications ranging from everyday disposable objects from bags, containers, packaging, cosmetics, hygiene products (towels, diapers, and handkerchiefs) to products that require high mechanical resistance, such as helmets for cyclists, and printed wiring boards (for electronics), and various car panels [38,39,40,41]. Furthermore, current research supports the application of PHBV in denitrification systems to eliminate the high concentration of nitrates in wastewater [42].

However, despite the great expectations generated by PHBV, its use remains limited due to its high production cost [7,43]. Thus, the international scientific community is focusing much effort on three main areas of research: finding new microbial strains capable of accumulating higher levels of PHBV [44], developing much more efficient fermentative routes with renewable sources as a substrate [45], and reducing the costs of the polymer extraction process [46].

## 2. PHBV: Chemical Structure and Properties

**Figure 2:**
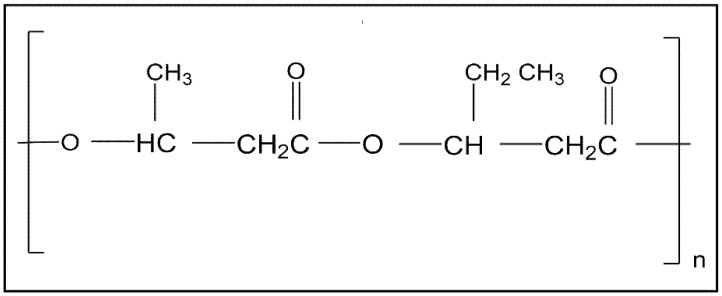
Chemical structure of the poly(3-hydroxybutyrate-*co*-3-hydroxyvalerate) copolymer.

Poly(3-hydroxybutyrate-*co*-3-hydroxyvalerate), also known as poly(3-hydroxybutyric acid-*co*-3-hydroxyvaleric acid) or just poly(hydroxybutyrate-*co*-hydroxyvalerate), abbreviated usually as PHBV or PHBHV, originates from the insertion of 3-hydroxyvalerate (HV) units to the PHB biopolymer. PHBV is an aliphatic polyester with the chemical structure shown in Figure 2. In addition, it is non-toxic, 100% biodegradable, biocompatible with many types of cells, characterised by its high degree of crystallinity, and it is resistant to ultraviolet radiation and acceptable amounts of alcohols, fats, and oils [17,47]. However, it is a rigid and rather brittle polymer, its melting temperature is lower than PHB, and it can be dissolved in chlorinated solvents [48].

PHBV also has excellent oxygen barrier properties, chemical inactivity, high viscosity in a liquid state, an aspect that is favourable in extrusion processes, and better mechanical properties, such as an increase in surface tension and greater flexibility compared to PHB [49]. Moreover, it has been developed on an industrial scale [21,23], and has recently attracted the attention of both industry and researchers as a promising material, due to its biotechnological potential, and its applicability in the medical, agricultural, and packaging fields [50,51]. Despite some of the improvements it offers over PHB (see Table 1), this polymer continues to exhibit high fragility, low impact resistance, considerable hydrophobicity and poor thermal stability compared to petroleum-based polymers [52].

Furthermore, the physical and mechanical properties of PHBV greatly depend on the 3HV content in the copolymer [54]. Thus, for example, the degradation rates of PHBV increase with the increase of 3HV fraction because the crystallinity of this biopolymer decreases with increasing 3HV content [55]. The melting point of this biopolymer decreases also with increasing the 3HV composition [56]. Therefore, it is important to select the PHBV with the desired 3HV content depending on its application.

PHBV was first commercialised by Imperial Chemical Industries (ICI) in 1990 [57]. However, its high current price is still the major obstacle to its widespread usage. For that reason, nowadays, the utilisation of this biopolymer is only economically feasible for certain applications.

### 2.1. Biodegradability

Biodegradability, which can be defined as the capacity of a substance to decompose through the activity of living organisms or as the gradual disintegration of a material in the presence of a specific biological activity, is of key importance in biomaterial applications. Thus, the material must have the capacity to degrade into products that can be easily metabolised by the organism once it has fulfilled the function for which it was created [58]. There are multiple factors that can affect biodegradation, among them the physical–chemical parameters of the ecosystem, such as temperature, pH, humidity, oxygen, light, etc.; microbial populations; physical and morphological properties of the PHB ,such as molecular weight, blends with others polymers, porosity, etc.; and the contact method between the biomaterial and the microorganisms [59].

The biodegradability property of PHBV has been demonstrated in soil, water, and compost [59,60]. Other studies detected no changes in molecular weight during the degradation of PHBV, and concluded that it occurred only on the surface of the polymer [61]. However, there was a decrease of molecular weight and a faster degradation of PHBV than PHB at high temperatures (40 °C). Furthermore, a recent study has reported that the biodegradation of PHBV was not affected by the addition of titanium dioxide nanoparticles as reinforcing agent [62]. PHBV reinforced with cellulose nanocrystals and zinc oxide (ZnO) showed, recently also, a moderate degradation rate of 9–15% after one week, in addition to showing excellent antibacterial activity [63]. Therefore, these results support the hypothesis that some physical, chemical, and/or biological properties of PHBV can be enhanced without hardly impairing its original biodegradability.

### 2.2. Molecular Weight

The molecular weight (MW) of a polymer is one of the most important factors in determining the properties of polymers, because it provides information about the length of the polymer chains and degree of polymerisation affecting the mechanical and biodegradability properties [2]. Thus, for example, at lower molecular weights, degradation will occur faster [64,65], and at molecular weights greater than 105 kDa, the material would not be suitable for use in biodegradable supports for medication administration [66].

The molecular weight of the polyhydroxyalkanoates depends on the type of microorganism and the extraction method used. Thus, Kim and colleagues obtained molecular weights less than 100,000 kDa for the PHBV copolymer from *Pseudomonas pulida*, while polymers with high molecular weights (from 1.5 to 1.8 × 10^6^ kDa) were obtained from the methanotrophic bacteria *Methylocystis* sp. [67]. On the other hand, the effect that extraction methods have on molecular weights has been described by different authors, particularly that the use of substances such as sodium hypochlorite and surfactants during the extraction of the polymer reduces the molecular weight by 30–50% [68,69]. Thus, an aqueous PHBV extraction process with thermal and enzymatic treatments produced a molecular weight of 6 × 10^5^ kDa, while the same extraction with solvents alone yielded an average of 1 × 10^6^ kDa [68].

## 3. Mechanical Reinforcement

The applications of many polymers, such as PHBV, is limited due to its poor mechanical properties compared to conventional polymers, such as lower impact resistance, reduced elongation at break, and fragility [70]. To address this problem, PHBV has been modified by crosslinking of this copolymer using dicumyl peroxide (DCP) as the initiator, to improve its mechanical properties [71]. However, the reinforcement approaches that have most often been conducted are the combinations of PHBV with other materials, such as polymers, natural fibres and many types of nanomaterials of different chemical nature, with the aim of expanding its applications.

### 3.1. Reinforcement with Other Polymers

One of the reinforcement strategies consists of mixing PHBV with other polymers. Thus, the results point to an improvement of mechanical properties, immiscibility, and degradation when mixing PHBV with variable proportions of polylactic acid (PLA), a thermoplastic biopolymer derived from lactic acid [72,73]. PHBV-based films prepared by mixing with a polythiophene carboxylate derivative, poly(3-thiophene ethyl acetate) (PTAcEt), exhibited great flexibility and resistance to handling [74]. Materials obtained from the blend of polyethylene (PE) with up to 30% PHBV exhibited mechanical properties, such as tensile strength, Young’s modulus, and elongation at break, comparable to those of commercial plastics. Moreover, the incorporation of PHBV reduced the rate of oxygen transmission, and increased the speed of water vapour transmission in comparison to that of pure PE, which are highly valued in the food packaging industry [75]. In addition, it has been reported that mixing 20–35% of PHBV with PLA is a suitable combination to achieve high barrier properties to both oxygen and water vapour, while maintaining the biocompatibility of the material [76]. However, the mixture of PHBV with PLA produces immiscible systems with minimal flexibility improvement [77]. Although, the effects of gamma radiation on the physical properties of these PHBV/PLA blends showed no significant changes in comparison with the decrease produced on both Young’s modulus and hardness of the pure PHBV and PLA polymers after 100 kGy of radiation [78].

Another procedure that can be utilised to improve polymer’s properties is the development of multilayer structures, where materials with different properties are combined in the same sheet [79]. This technique could also overcome PHBV’s incompatibility for being immiscible with proteins and polysaccharides, due to the low interfacial adhesion between the phases of the mixture [80]. The blending of PHBV with starch, one of the most abundant and economic biopolymers, obtained from renewable sources and with low oxygen permeability [81], gives rise to composite materials with better mechanical properties and a low permeability to water vapour, making it more suitable as a material for the conservation and packaging of food [82]. It is important to mention that the incorporation of other compounds, such as plasticisers (e.g., polyethylene glycol and lauric acid) into PHBV films, can reduce the stiffness and breaking strength of the plasticised films, in comparison to pure PHBV films [83].

On the other hand, brittle biomaterial hydrogels such as poly(hydroxyethyl methacrylate) (PHEMA) can be reinforced with PHBV by photopolymerisation of hydroxyethyl methacrylate (HEMA) in the presence of PHBV to form IPNs with enhanced mechanical properties [84]. 

### 3.2. Reinforcement with Natural Fibers

The incorporation of vegetal fibres into biodegradable plastic compounds of natural origin is acquiring a greater relevance every day [85]. These materials, in addition to being obtained from sustainable renewable sources, produce waste products that can be assimilated and degraded by a great variety of microorganisms, thereby avoiding the harmful accumulation of waste in the environment. Achieving the development of these biomaterials would be a step forward in the replacement of petroleum-based plastics [86,87]. Moreover, the search for the production of ecological composite biomaterials based on renewable resources has been the driving force for a variety of studies aimed at reinforcing PHBV with natural fibres. Thus, PHBV-based biocomposites containing 10–40% *w*/*w* of the maple wood fibre have been developed [88]. When evaluating the properties of this material, it was concluded that the tensile and flexural modulus of the materials reinforced with 40% *w*/*w* of fibre was improved by 167%, compared to pure PHBV. The values of the storage modulus of these biocomposites also showed an increasing tendency in relation to the pure polymer: the deflection temperature was increased by 21%, while the linear thermal coefficient for expansion of the pure PHBV compound was reduced by 18%.

Bamboo fibres have also shown to be a valuable reinforcing material for PHBV at 30–40% *w*/*w* [89]. Thus, the tensile modulus of the materials with 40% *w*/*w* of bamboo fibre increased by 175% with respect to pure PHBV, and the heat deflection temperature increased to 9 °C. However, it is important to achieve sufficient interfacial interaction between the bamboo fibres and the polymer matrix because otherwise, the tension strength of PHBV can decrease [90].

Therefore, the reinforcement of PHBV with natural fibres results in being a good option, due to the biocompatibility and biodegradability of the obtained composite materials, which are very promising for advanced applications such as sustainable food packaging [91,92].

### 3.3. Reinforcement with Nanomaterials

Nanotechnology is extensively used to define the sciences and techniques that are applied at the nanoscale and is characterised by being an essentially multidisciplinary field that offers a wide range of solutions to different fields and sectors, both scientific and technological [93]. Thus, another option for improving the mechanical properties of PHBV consists of producing nanocomposites with nanoparticles as reinforcing agents that can be also biodegradable and come from renewable sources [94]. The most promising nanomaterials utilised so far are carbon nanomaterials, such as graphene, graphene oxide, reduced graphene oxide (rGO), carbon nanotubes (CNTs), and carbon nanofibers, and other nanomaterials of very different chemical nature, such as nanocellulose, nanoclay, and nanometals.

#### 3.3.1. Carbon Nanomaterials

One of the carbon nanomaterials that can be used to improve PHBV properties is graphene, which is a 2D atomic sheet formed by carbon atoms arranged evenly in the shape of hexagons, with excellent thermal and electrical conductivity [95]. Furthermore, graphene is a very stable compound and it has been shown to be biocompatible, improving the cell adhesion of osteoblasts and mesenchymal cells [96]. It is an excellent material whose resistance is two hundred times superior to that of steel, and which has outstanding properties of elasticity, flexibility, as well as great mechanical properties, making graphene an excellent reinforcement option for materials with poor mechanical properties [97].

The graphene family consists of materials arising from physical or chemical modifications of graphene. For example, oxidation or reduction treatments produce graphene oxide or reduced graphene oxide respectively. For certain applications, it is of great importance to convert graphene into other compounds because, for example, pure graphene has shown a certain cytotoxicity, which could induce cellular apoptosis and a decrease in cell adhesion [98]. Furthermore, this material has exhibited very significant antibacterial activity against a variety of bacterial species, although the mechanism of action is unclear [99]. Thus, graphene oxide provides the possibility of reinforcing PHBV, acting as a nucleating agent for crystallization, which substantially improves the mechanical properties of composite materials and increases the temperature of maximum degradation. Furthermore, the presence of GO does not interfere with the biodegradability of PHBV, although it could restrict the mobility of PHBV chains in the crystal growth process [100]. PHBV have also been reinforced with the inclusion of magnetite nanoparticles and reduced graphene oxide [101]. This study revealed a clear improvement of the mechanical strength of the nanocomposite, in comparison to the pure PHBV copolymer, very promising for tissue engineering applications.

Another important carbon nanomaterial, discovered in 1991 by Sumio Iijima, are the carbon nanotubes (CNTs) [102]. These nanomaterials are one-dimensional systems in the form of single-layer or multilayer CNTs, named as single-wall carbon nanotubes (SWCNTs) and multi-wall carbon nanotubes (MWCNTs) respectively, with exceptional mechanical, thermal, electrical, and electronic properties, which allow great applicability in various fields of nanotechnology [103,104]. Thus, CNTs have been inserted into the PHBV polymer matrix to enhance its low mechanical properties. In addition, the nanocomposites of PHBV with MWCNTs, which in general provide electrical conductivity to the nanocomposite, showed an effective improvement in crystallization and nucleation over pure PHBV, demonstrating an significant increase in mechanical properties [105,106,107,108]. The tensile strength and the Young’s modulus of the PHBV film with 7% *w*/*w* of MWCNTs improved by 88% and 172% in comparison with those of the pure polymer [109]. Furthermore, the composite biomaterials showed lower water absorption and water-vapour permeability.

Carbon nanofibers (CNFs) like CNTs can be used to enhance the conductivity, thermal, mechanical, and to enhance gas barrier properties of thermoplastic biopolyesters, such as PHBV and polycarpolactone (PCL) [110]. CNFs, sometimes referred to as carbon nanofilaments or graphitic nanofibers, are graphitic carbon structures in which the carbon atoms are grouped into filiform structures with excellent mechanical, electrical, and thermal properties, suitable for advanced applications, such as regenerative medicine [111]. Furthermore, CNFs are much more economical than CNTs [112]. 

#### 3.3.2. Nanocellulose

**Figure 3:**
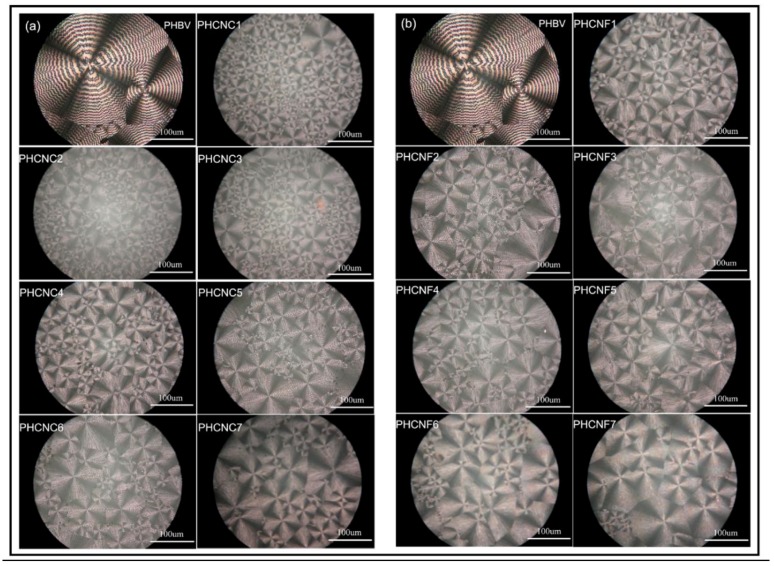
Polarised optical microscope photographs: (**a**) cellulose nanocrystal (CNC)/PHBV composites, and (**b**) cellulose nanofibril (CNF)/PHBV composites. Scale bar represents 100 µm. Reproduced with permission from Ref. [9].

Various studies point to nanocellulose as an excellent choice for the reinforcement of PHBV, due to its unique properties, among which the following stand out: a high Young’s modulus, dimensional stability, a low coefficient of thermal expansion, exceptional reinforcement potential, and transparency [113]. Besides its mechanical properties, nanocellulose has low density, and the presence of surface hydroxyl groups facilitates the anchorage of specific chemical groups that improve its compatibility with other polymers [114]. Nanocellulose can be presented in two morphology types: cellulose nanocrystals (CNCs) and cellulose nanofibrils (CNFs). Both types improve the thermal stability of PHBV and show a reinforcement effect on the biopolymer, however, the CNCs act as a better nucleation agent because the crystals are distributed homogeneously in the polymer matrix [115]. The nucleation effect of CNCs and CNFs on crystallization behaviour of PHBV composites was studied by polarised optical microscope (POM) (see Figure 3).

With incorporation of CNCs, the number of PHBV spherulites increased reducing dramatically their sizes (Figure 3a), confirming the nucleation effect of CNCs for crystallization of PHBV. CNFs could also act a nucleation agent in PHBV (Figure 3b). However, the optimal mechanical properties of PHBV were found with the addition of 1% *w*/*w* of CNCs.

Recent studies have demonstrated that the composite materials of CNCs and PHBV showed a decrease in crystallinity with respect to pure PHBV, and that the measures of the contact angles indicated an increase in the hydrophilicity of the nanocomposites [116,117]. The significant improvements of mechanical properties were attributed to the hydrogen bonding interactions between the cellulose nanocrystals and the PHBV matrix [118].

The addition of nanofibrillated cellulose (NFC) as reinforcement agent to PHBV resulted in completely biodegradable composite materials (PHBV/NFC) with an increase of almost twice the stress–strain modulus [119]. However, a high NFC content can lead to further thermal degradation of the PHBV matrix.

#### 3.3.3. Nanoclays

Nanocomposites that contain clays are environmentally friendly, and can greatly enhance some material properties, such as thermal stability, conductivity, mechanical properties, and gas and vapour barrier properties [94]. Thus, some studies indicate that the incorporation of nanoclays (NC) into the polymeric matrix of PHBV plays an important role in the dispersion of clay, which is crucial for improving the material’s performance, such as the increase of the Young’s modulus [120]. In addition, it has been demonstrated that well dispersed organomodified clays improve the thermal and dynamic mechanical properties of the pure PHBV matrix [121].

For example, as a crystalline nucleating agent, NC, significantly enhanced the crystallinity of PHBV in a blend of PLA and PHBV, thus leading to a relatively high modulus for both solid and microcellular specimens. However, the incorporation of NC had less of an effect on the tensile strength and strain-at-break [122].

#### 3.3.4. Nanometals

Tungsten disulphide (WS_2_) is a material with excellent electrical, optical and tribological properties [123,124]. Thus, a significant enhancement in the thermal stability of the PHBV polymer, an efficient nucleation effect, and a good dispersion can be achieved when blending this biopolymer with WS_2_, in comparison with that obtained with other reinforcement nanomaterials or specific nucleating agents, which provide these nanocomposites a broad range of applications in the field of sustainability and biomedicine [125]. In addition, very recent nanocomposites of PHBV and low loadings of tungsten disulphide inorganic nanotubes (INT-WS_2_) increased the crystallization rates of PHBV, due to the high nucleation efficiency of INT-WS_2_ on the crystallization of PHBV [126].

Boron nitride (BN) is a binary compound consisting of equal proportions of boron (B) and nitrogen (N) distributed with an atomic structure similar to diamond [127]. Thus, BN films have excellent characteristics, such as high hardness, resistance to chemical attack, insulating behaviour, and high transparency.

PHBV has been reinforced with BN particles and compared to the reinforcement produced by the addition of other nanomaterials, such as aluminium borate whiskers (ABOw) and CNTs [128]. The analysis of this study showed that these three types of nanoreinforcements increased the decomposition temperatures, thus improving the resistance to the degradation of the nanocomposite at elevated temperatures. The nanoparticles of ABOw showed the most significant increase, in addition to improving the mechanical performance of pure PHBV [128]. The incorporation of BN particles into PHBV via melt processing decreased permeability and increased crystallinity compared to pure PHBV. As a result, the barrier properties of the nanomaterial were improved [129].

Titanium dioxide (TiO_2_) is another nanomaterial that has been utilised to reinforce PHBV [62]. Furthermore, these nanoparticles did not affect the high rate of biodegradation of the PHBV biopolymer matrix, and thus, produced valuable nanocomposites with applications as biodegradable materials.

**Figure 4:**
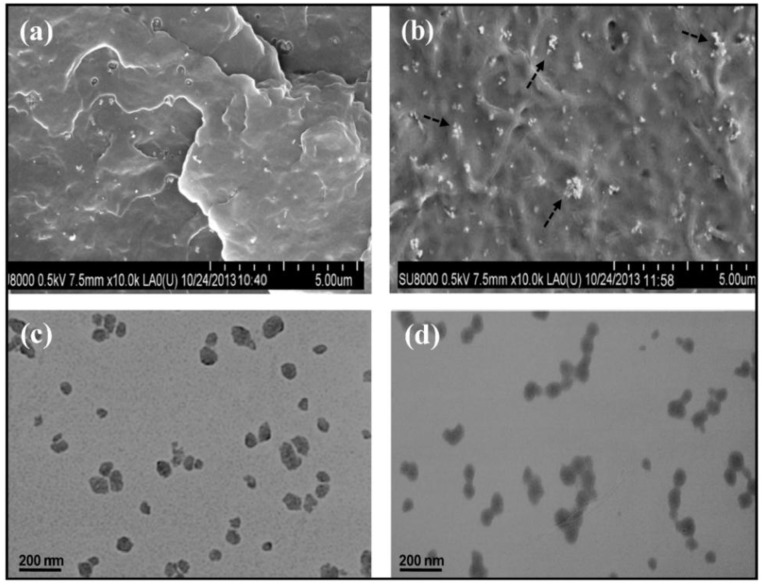
Scanning electron micrographs from fractured surfaces of PHBV/ZnO nanocomposites with (**a**) 1% *w*/*w* and (**b**) 8% *w*/*w* ZnO loading. The arrows in b point out small nanoparticle aggregates. Transmission electron micrographs of PHBV/ZnO nanocomposites with (**c**) 4% *w*/*w* and (**d**) 8% *w*/*w* ZnO content. Reproduced with permission from Ref. [62].

ZnO nanoparticles have been successfully dispersed within the PHBV polymer matrix to produce nanocomposites with superior stiffness, strength, toughness, and glass transition temperature [130]. Moreover, these sustainable nanomaterials possess intensic antimicrobial activity, which is very desirable in the containers for beverage and food products in the food packaging industry. Scanning electron microscopy (SEM) images from cryofractured surfaces of PHBV nanocomposites with 1 and 8% *w*/*w* ZnO are shown in Figure 4a,b respectively. The white dots in the SEM micrographs correspond to the nanoparticles that present quasi-spherical shape. Transmission electron microscopy (TEM) was also performed to assess the state of ZnO dispersion within the PHBV matrix, and the micrographs of nanocomposites with 4 and 8% *w*/*w* loading are shown in Figure 4c,d respectively.

## 4. Improvement of Thermal Properties

The melting point and glass transition temperature of PHBV is about 153 °C and −1 °C, respectively (see Table 1). Therefore, this biopolymer behaves as a rubber at room temperature and it is unstable above 160 °C during melt processing, which limits its practical applications as a commodity material [131]. Furthermore, it is of note that these thermal properties can be significantly affected by the addition of plasticisers [132], radiation such as ^60^Co γ-radiation [133], and degree of crystallinity [134].

Therefore, suitable enhancement strategies have been developed in order to improve the thermal properties of this biopolymer in the last decade. For example, the low thermal resistance of PHBV has been minimised, combining blending with polymers such as poly(ε-caprolactone) (PCL) and small amounts of TiO_2_ nanoparticles [135]. The addition of only 1% *w*/*w* of TiO_2_ nanoparticles significantly improved the thermal stability of both polymers in the blend. Other blends, such as that of PHBV and poly(butylene succinate) (PBS) at different weight ratios (80, 50, and 20% *w*/*w*) also produced materials with better thermal stability and fire reaction in comparison with pure PHBV [136].

The effects of adding nanoparticles such as nanoclay (NC) on PHBV/poly(butylene adipate-*co*-terephthalate) (PBAT)/silane-treated-recycled wood fibre (RWF) composite did not show significant changes of mechanical properties at a low filling content (2%) [137]. However, it did enhance significantly its thermal stability. Poly(3-thiophene ethyl acetate)—PTAcEt, added to the PHBV matrix in an amount of up to 12%, caused also improvements in thermal stability and in the degree of bioactive matrix crystallinity [74], and the addition of plasticisers, such as polyethylene glycol, resulted in plasticised films that had high heat resistance and the ability to partially mitigate the effects of aging on the material [83].

Other nanoparticles, such as zinc oxide (ZnO) blended with PHBV, significantly improved the thermal stability and the optical and barrier properties of the polymer, which are very important in in the food packaging industry [138].

Combining PHBV with cellulose nanocrystals caused the formation of strong intermolecular interactions of hydrogen bonds, due to the excellent dispersion of the cellulose nanocrystals in the PHBV matrix, achieving improvements in thermal stability [118].

Another alternative to enhance the thermal properties of PHBV consists of the incorporation of carbon nanomaterials, such as graphene nanosheets (GNS) or CNTs. Thus, however, the thermal stability of PHBV was enhanced significantly with a low load of GNS [139]. Transparent bionanocomposite films of PHBV, reinforced with 1–10 wt % of PHBV-grafted multi-walled carbon nanotubes (PHBV-g-MWCNTs) improved, also, the thermal stability and the mechanical, barrier, and migration properties of PHBV [109]. These excellent results can be attributed to the fact that the PHBV-g-MWCNTs were uniformly dispersed throughout the PHBV matrix in the nanocomposite films.

## 5. Enhancement of Wettability

Material wettability or hydrophilicity constitutes a critical factor for adequate cell proliferation and tissue regeneration [140]. Despite the good biocompatibility properties of PHBV, the hydrophobic nature of its surface makes it very unlikely for cell adhesion and proliferation. To overcome these limitations, the surface of this biomaterial must be modified to promote cell adhesion and proliferation [141]. Therefore, there are several techniques that are capable to cause changes in the chemical or biological properties and physical appearance of the PHBV surface. These methods include treatment with plasma, chemical treatment and ultraviolet radiation.

### 5.1. Treatment with Plasma

Surface treatment with plasma gas (N_2_, O_2_, NH_3_, Ar) [142] is one of the methods that can be used to improve the hydrophilicity of hydrophobic polymer, such as PHBV. Plasma is a gaseous mixture of electrons, radicals, ions, and excited molecular states produced by inelastic collisions between high-energy electrons and atoms or molecules in their ground state [143]. It is an economical and viable technique commonly utilised in many industrial production processes to modify the surface roughness and hydrophilicity of materials. Furthermore, this technique can be modified by regulating the type of gas used and the treatment conditions and plasma parameters [144]. Thus, PHBV films treated with N_2_ plasma exhibited higher surface roughness than untreated films, and after 10 s of plasma treatment, the water contact angle value decreased from 75.2° to 51.5° [145]. In another study, the surfaces of PHBV treated with N_2_ plasma showed greater cell affinity, growth, and proliferation in comparison to the untreated surfaces [146]. These films also showed greater interaction and cell adhesion than the films treated with O_2_ plasma, demonstrating that surfaces that are moderately hydrophilic favour cell proliferation and adhesion more than hydrophobic or highly hydrophilic surfaces.

Nanofiber mats of PHBV have been treated with plasma (O_2_ or N_2_) and by immobilisation with silk fibroin to enhance their hydrophilicity and biocompatibility, respectively [147]. These results indicated an increase in hydrophilicity without alteration of morphology and the nanofiber mats treated in the presence of N_2_ showed the highest cellular viability.

Very recently, both plasma treated PHB and PHBV films have shown increase of hydrophilicity compared to that of the untreated films. However, an in vitro experiment of mouse adipose-derived stem cells showed better cell growth and adhesion on the surface of plasma-treated PHBV film [148].

### 5.2. Chemical Treatment

Chemical treatments can break ester bonds to produce functional groups, thus altering the surface, the chemical state, and the morphology, among other aspects of PHBV. Films of PHBV treated with KOH showed a decrease in contact angle values with water and had an adsorption of albumin, collagen, and fibronectin almost 100 times higher than the unmodified PHBV films [141]. Short chemical treatments with ethylenediamine decreased the hydrophobicity of PHBV without causing significant alterations in its mechanical properties [149].

The hydrophilicity of PHBV magnetic microspheres significantly increased after the modification of the surface with lauric acid [150]. Additionally, the microspheres did not show cytotoxicity, and thus, they are excellent candidates for biomedical applications, such as the controlled administration of drugs and for resonance studies.

In the same way, the modification of PHBV with poly(ε-caprolactone) (PCL) in the presence of triethyl citrate (TEC) and dicumyl peroxide (DCP) used as a plasticiser and grafting agent, respectively, caused an increase in the hydrophilicity of the PHBV blends [151].

Another alternative enhancement approach to improve its cell compatibility consisted of covalent immobilisation of collagen onto PHBV films [152]. Chondrocytes showed better cell adhesion and proliferation on the collagen-modified PHBV film than on the pure PHBV film, indicating that this biomaterial could be very successful in cartilage tissue engineering.

### 5.3. Ultraviolet Radiation

Ultraviolet (UV) radiation is a very economical, rapid, easy and reproducible alternative method that has a significant effect at the superficial level without causing alterations in the internal structure of the polymer [153]. Thus, PHBV films blended with vinylpyrrolidone and irradiated with UV radiation using a photosensitiser (benzophenone) significantly improved hydrophilicity [141]. Acrylamine monomers have also been grafted on the surface of PHBV scaffolds via UV radiation to achieve more hydrophilicity and exhibit greater cell adhesion and proliferation than on the pure PHBV scaffolds [154,155].

## 6. Water Absorption Properties

The hydrophobic properties of PHBV can be modified by grafting of chemical compounds, such as, for example, maleic anhydride, into the PHBV matrix, and thus enhance the barrier properties against oxygen and water vapour [156]. Plasticisers, such as polyethylene glycol, lauric acid, and stearic acid, can be incorporated into PHBV films at 10% *w*/*w* to increase the films’ water sorption capacity and solubility, as well as their water vapour permeability [83].

Other modification strategies consist of blending PHBV with silane fibres and clay particles [157]. This modification increased the hardness and the water resistance of PHBV. Furthermore, hydrophilic fillers, such as straw fibres, can be utilised to enhance the water vapour transfer (sorption and diffusion) properties of PHBV [158].

Another study showed that by introducing nanohydroxyapatite and modulating its loading level in PHBV, the water absorption process of the biopolyester-based biomaterials can be controlled as required, which is very useful for designing new medical devices or implants [159].

## 7. Antimicrobial Activity

The development of new advanced materials that are able to hinder or inhibit microbial growth, as an alternative strategy against the global health threat of microbial resistance [160], is an arduous task that many scientists are currently undertaking.

Despite the excellent properties of PHBV, the lack of antimicrobial activity of this biopolymer [161] restricts its potential applications. Thus, to solve this problem, the incorporation of additives with proven antimicrobial activity is considered a feasible option for obtaining biodegradable antimicrobial materials [162]. Thus, some essential oils, especially oregano, *Origanum vulgare* [163], and clove, *Syzygium aromaticum* [164], are among the natural substances with notable microbicidal activity. The antimicrobial activity of oregano oil is attributed to its two main components, carvacrol and thymol, which are capable of altering the permeability of bacterial membranes, especially those that are Gram-positive, producing an imbalance in cellular homeostasis [163]. Thus, oregano essential oil has been shown effective against *Staphylococcus aureus*, *Escherichia coli*, *Pseudomonas aeruginosa*, *Listeria monocytogenes*, and *Salmonella enteriditis* [165], and against mycotoxigenic fungi, such as *Aspergillus, Penicillium*, and *Fusarium* [166,167]. Similarly, clove essential oil has been shown to have antimicrobial activity against microorganisms, such as *S. aureus*, *Bacillus cereus*, *E. coli*, and *L. monocytogenes*, mainly due to the presence of eugenol as the main component of this oil [164]. The drawback of incorporating essential oils as active compounds in biodegradable films is the loss of volatiles during the film production process. Thus, a successful method to overcome this problem consists of spraying only one side of a film and subsequently thermo-compressing two PHBV films together, obtaining a antimicrobial bilayer film with the active compounds at the interface [168].

Several studies have been focused on another strategy consisting of blending PHBV with alternative materials that have potential antimicrobial effects, such as silver nanoparticles [169]. The results of this study showed high antibacterial activity against strains of *S. aureus* and *Klebsiella pneumoniae*. Moreover, the scaffolds produced with this composition were not cytotoxic, and showed good in vitro cellular compatibility. In other similar studies, the nanocomposites of PHBV with silver nanoparticles also showed strong in vitro antibacterial activity against other important bacteria, such as *Salmonella enterica* and *L. monocytogenes* [161,170].

PHBV composite materials developed as bilayer structures containing antimicrobial oxides such as copper oxide (CuO) nanoparticles via compression moulding showed significant bactericidal and virucidal performance against the foodborne pathogens *S. enterica*, *L. monocytogenes*, and *Murine norovirus* [171]. In the same way, PHBV/ZnO films have shown also effective and prolonged antibacterial activity against *L. monocytogenes* [138] and other human pathogenic bacteria, such as *E. coli* and *S. aureus* [130,172].

PHBV membranes prepared by treatment with ozone, followed by grafting with acrylic acid, subsequent grafting with chitosan (CS) or chitooligosaccharide (COS), and immobilisation of hyaluronic acid (HAc) onto the CS- or COS-grafting membranes, showed that the antibacterial activity of CS and COS against *S. aureus*, *E. coli*, and *P. aeruginosa* was preserved after HAc immobilisation [173]. Among them, CS-grafted PHBV membrane showed higher antibacterial activity than COS-grafted PHBV membrane.

**Figure 5:**
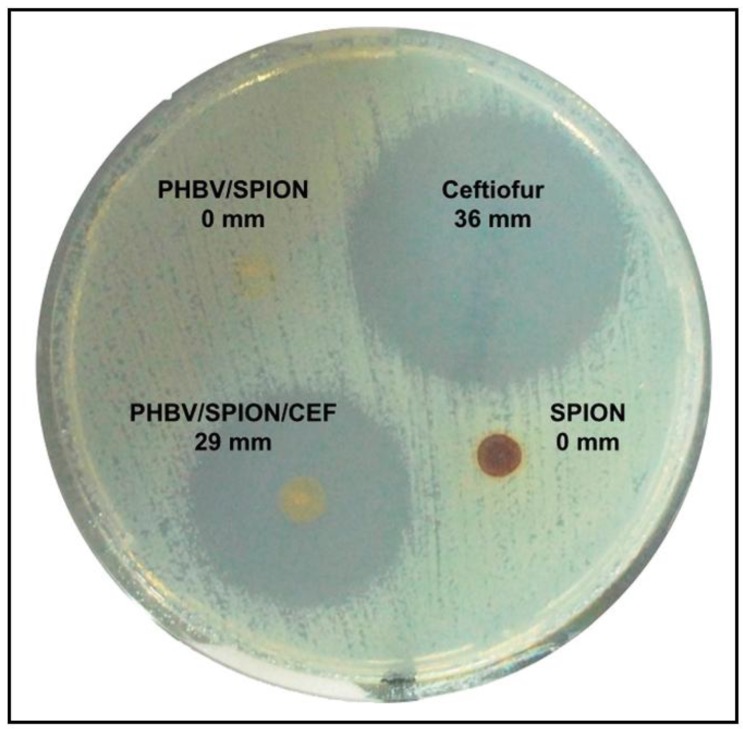
Determination of the antimicrobial activity of PHBV nanoparticles, ceftiofur and superparamagnetic iron oxide nanoparticles (SPIONs) against *Escherichia coli* (ATCC 25922) through the agar diffusion test at 24 h of incubation. Reproduced with permission from Ref. [174].

A recent development of polymeric nanoparticles based on PHBV functionalised with superparamagnetic iron oxide nanoparticles (SPIONs) and/or the antibiotic ceftiofur (CEF) (PHBV/SPION/CEF) have shown to be very promising for many biomedical applications, due to their potential use for the treatment of bacterial infections [174] (see Figure 5).

The results of this study of the PHBV/CEF/SPION nanoparticles against *E. coli* by the agar diffusion test showed positive antibacterial activity (inhibition halo of 29 mm) close to that of free ceftiofur (halo of 36 mm). Furthermore, the PHBV/SPION and SPION samples did not show any bacterial growth inhibition halo, as expected.

Due to the antimicrobial activity of graphene and its derivatives [99], the addition of these carbon nanomaterials have also been evaluated in order to enhance the antimicrobial properties of PHBV. Thus, non-toxic, antimicrobial, biocompatible, and biodegradable magnetic Fe_3_O_4_/rGO-g-PHBV composite-based porous 3D scaffolds have been developed. In the same way, GO have been recently utilised to improve the mechanical strength, surface area, and antibacterial properties of nanofibrous PHBV/collagen/GO scaffolds for wound coverage [175]. The incorporation of GO in the PHBV matrix increased the mechanical strength of the scaffold, in addition to the antibacterial activity against *E. coli* and *S. aureus*.

## 8. Development of Porous PHBV Scaffolds

Current tissue engineering strategies focus on the reconstruction and regeneration of damaged tissues by 3D cell culture in porous scaffold biomaterials. To restore the functionality of an organ, the presence of a scaffold is essential as extracellular matrix for cell colonisation, migration, growth, and differentiation, until the tissues are restored or regenerated completely [176]. In order to develop porous scaffolds containing biological agents, it is necessary to provide adequate conditions that not only allow tissue regeneration, but also prevent the recurrence of tumours after surgical operations [177].

The scaffold fabrication technique must be selected within a wide range of alternative methods that have been developed in the last decades: polymerisation in solution [178,179,180,181], leaching [182,183,184], electrospinning [175,177,185], and 3D printing [186,187,188,189], among others [190].

### 8.1. Electrospun PHBV Scaffolds

**Figure 6:**
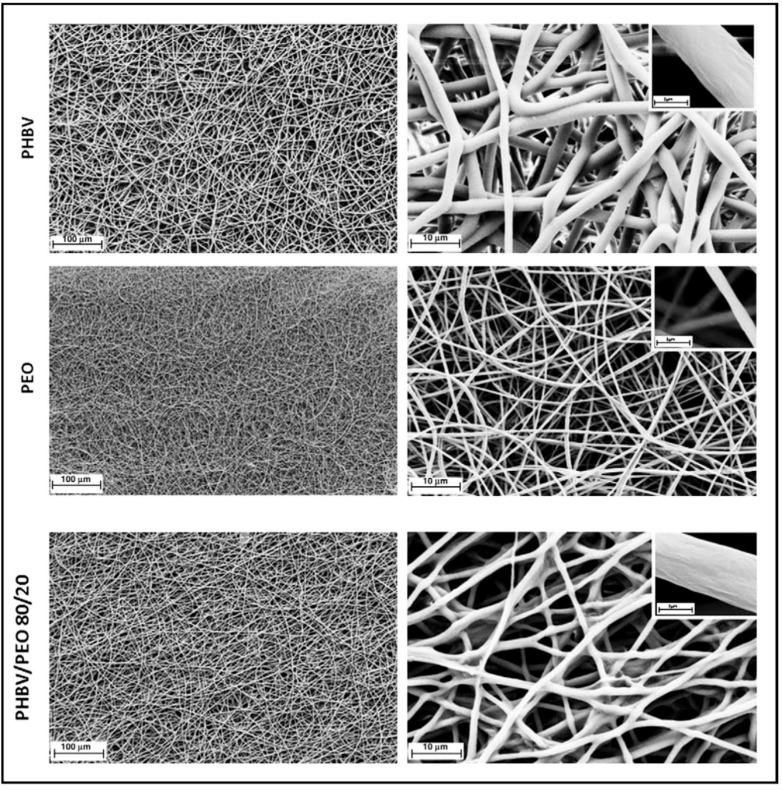
Scanning electron micrographs of electrospun scaffolds of poly(3-hydroxybutyrate-*co*-3-hydroxyvalerate) (PHBV), polyethylene oxide (PEO) and PHBV/PEO 80/20. Reproduced with permission from Ref. [185].

In the biomedical field of tissue engineering, nanofibrous scaffolds have shown better biological behaviour than microfibrous scaffolds. For example, promising scaffolds composed of PHBV/polyethylene oxide (PEO) polymer fibres of nanometric size have been prepared by means of coaxial stretching electrospinning of a viscoelastic solution [185] (see Figure 6).

These fibres have diameters ranging from submicron to nanometer, ranges in which it is possible to find unique characteristics, such as a very large surface area in relation to volume [191].

As it was mentioned in the previous section, antimicrobial porous scaffolds of PHBV nanofibers using the electrospinning method with collagen and reinforced with graphene oxide (GO) have been recently developed [175]. The combination of collagen and GO with PHBV has well-balanced properties, and can be used in biomedical applications, due to the structural resemblance of the native extracellular matrix and high porosity. Furthermore, collagen enhanced the cell proliferation capacity of the nanofibers in comparison with the samples of PHBV with GO and pure PHBV.

An alternative technique for fabricating nanofibers with a more effective way of reducing PHBV fibre diameters to the nanosize range is based on coaxial electrospinning [191]. In order to obtain PHBV nanofibers, the structured core and shell fibres were first fabricated by coaxial electrospinning, with PHBV as the core and chitosan as the shell. After that step, the chitosan shell was then removed by washing the electrospun frames with water, which led to the formation of nanofibrous PHBV scaffolds.

Nanofibers composed of PHBV, dextran, laminarin, hydrolysed dextran, and hydrolysed laminarin, using an electrospinning process, exhibited a completely interconnected pore structure [177]. Furthermore, the hydrophilicity was enhanced by the addition of low molecular weight glucans. Cell viability tests on the resulting scaffolds revealed adequate proliferation of skin fibroblasts, due to the antioxidant activity and the hydrophilicity of the material.

In addition, recent advances have shown that scaffolds composed of electrospun nanofibers can possess significant control over the kinetics of drug release, and great potential in maintaining therapeutic doses over a longer period compared to other drug-releasing scaffolds [192].

### 8.2. PHBV Scaffolds by Leaching

Leaching consists of a process in which a solvent gets in contact with a solid mixture, resulting in the dissolution of one or more soluble components [193].

Thus, porous PHBV scaffolds mixed with icariin, a bioactive ingredient of the traditional Chinese medicine, was fabricated with this porous scaffolding leaching method [194]. The resultant porous matrix showed potential commercial use for tissue engineering because it exhibited a very high porosity (88.80%), and appropriate mechanical properties for cell culture and proliferation.

PHBV scaffolds have also been built, integrating nanoparticles of beta-Ca_2_SiO_4_ into the main PHBV polymer chains, through a particular leaching method, to generate an interconnected porous structure [184]. Such scaffolds, with respect to those of pure PHBV, demonstrated increased hydrophilicity and cell adhesion of MG-63 cells similar to human osteoblasts. In addition, they induced early differentiation through the promotion of alkaline phosphatase transcription.

Other porous scaffolds composed of PHBV, hydroxyapatite (HAp), and type I collagen were fabricated using a hot press machine and salt leaching [195]. The results showed that PHBV/HAp/Col composite scaffolds have better cell adhesion and significantly higher proliferation and differentiation than PHBV/HAp composite scaffolds and pure PHBV scaffolds.

Other porous 3D PHBV scaffolding approaches with carrageenan as filler, extracted from the *Kappaphycus alvarezii* red algae, utilised the solvent casting particulate leaching (SCPL) method [196]. The results of this work showed that these structures were suitable for long-term cell cultures (>2 weeks). Moreover, the use of PHBV with carrageenan as filler exhibited great potential for applications in tissue engineering.

### 8.3. PHBV Scaffolds by 3D Printing

3D printing techniques, such as selective laser sintering (SLS) and fused deposition modelling (FDM), are approaches to fabricate complex shapes for scaffolds’ production from a computer aided design (CAD) file [197]. In these technological approaches, 3D scaffolds are produced layer-by-layer, without any part-specific tooling or dies [198,199]. Over the last few years, 3D printing has evolved from its initial application, as pre-surgical visualisation models and tool moulds, to the creation of scaffolds for tissue engineering, diagnostic platforms, and drug delivery systems [200]. Thus, three-dimensional mesoporous bioactive glass and poly(3-hydroxybutyrate-*co*-3-hydroxyhexanoate) composite scaffolds have been synthesised with well-defined pore structures and high compressive strength by a 3D printing technique [186]. 

**Figure 7:**
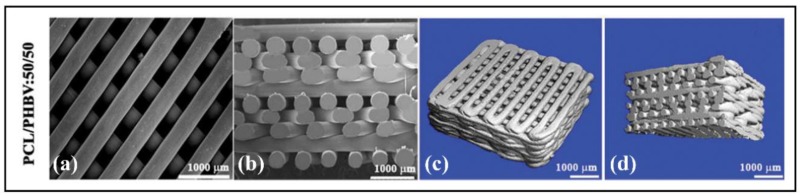
Microstructures of PCL/PHBV:50/50. Scaffolds examined by SEM microscopy (**a**,**b**) and by microcomputerised tomography (µ-CT) (**c**,**d**). Reproduced with permission from Ref. [187].

The FDM is a rapid prototyping (RP) technology that can be used to fabricate novel scaffolds with honeycomb-like pattern, high porous interconnection, and controllable porosity [201]. Thus, porous composite biodegradable PCL/PHBV scaffolds were fabricated by FDM (see Figure 7) and exhibited enhanced surface roughness, wettability, and hydrophilicity after low pressure oxygen plasma treatment [187]. In addition, the results of this study showed that the compression resistance and cell proliferation increased with increasing the PHBV content.

The SLS technology is a mature RP technique that is able to construct sophisticated porous structures of complex shape with good quality and high potential use in some tissue engineering applications [202]. Thus, for example, calcium phosphate (Ca-P)/PHBV scaffolds have been fabricated by the SLS technology for bone tissue engineering applications [188], because Ca-P/PHBV nanocomposite scaffolds produced by this 3D printing technique had shown better mechanical properties than neat PHBV scaffolds [189]. In addition, porous PHBV/calcium silicate (CS) composite scaffolds fabricated via SLS have shown enhanced bioactivity and mechanical properties due to the CS addition [203].

Furthermore, these 3D printing techniques can be perfectly combined with any of the methods for fabricating PHBV-based scaffolds mentioned before in this section. Thus, for example, the combination of electrospinning and 3D printing was used to produce biodegradable synthetic PHBV-based perfusable vascular networks [204].

## 9. Conclusions and Future Perspectives

In conclusion, the PHBV microbial biopolymer is a renewable natural material that is very promising for a wide range of applications, due to its excellent innate properties, such as biodegradability and biocompatibility. However, many of its physical and mechanical properties greatly depend on the 3HV content, and are, in general, very poor for certain applications. In order to extend its potential applications, properties such as mechanical strength, water sorption and diffusion, electrical and/or thermal properties, antimicrobial activity, wettability, biological properties, and porosity, can be enhanced by following diverse strategies developed so far, and are described in this review. Thus, PHBV can be modified by combination with other materials of different chemical nature, such as other polymers, natural fibres, carbon nanomaterials such as graphene and its derivatives, nanocellulose, nanoclays, and nanometals, to produce advanced composites. Furthermore, this biodegradable polymer shows excellent properties to be used in the production of porous scaffolds for tissue engineering, and the most important fabrication methods of PHBV scaffolds were described. However, it is of note that PHBV produced by Gram-negative bacteria might contain high levels of endotoxins, which can cause inflammatory, pyrogenic, and other reactions in biomedical applications, if they are not well extracted. Another pending task so that PHBV becomes a real candidate for substituting conventional plastics consists of reducing its current high production cost, which limits its use for only certain applications. Even though many research groups are doing much effort on finding new microbial strains capable of accumulating higher levels of PHBV, developing much more efficient fermentative routes or reducing the costs of the polymer extraction process, the current price of this biopolymer is a good reason to discourage researchers in this field, and is still the major obstacle to its widespread usage. Therefore, much research needs to be conducted still in this research line, because there is a critical need to develop sustainable and affordable solutions in the face of the environmental devastation caused by petroleum-derived polymer waste contamination. Nevertheless, the enhanced strategies presented here are able to produce advanced biodegradable PHBV-based materials with improved properties as an outstanding solution for many current advanced applications. In addition, these rapidly progressing enhancement approaches open up new exciting opportunities in many important research fields, such as tissue engineering, biomaterial science, and biodegradable packaging.

## Figures and Tables

**Table 1:** Physical properties of polyhydroxybutyrate (PHB) and poly(3-hydroxybutyrate-*co*-3-hydroxyvalerate) (PHBV). Reproduced with permission from Ref. [53].

Properties	PHB	PHBV
Density (g/cm^3^)	1.25	1.25
Elasticity modulus (GPa)	0.93	2.38
Traction Resistance (MPa)	21	25.9
Elongation (%)	5.2–8.4	1.4
Fusion temperature (°C)	161	153
Glass transition temperature (°C)	−10	−1

## Abbreviations

PHAs	polyhydroxyalkanoates
HA	hydroxyalkanoate
PHB	polyhydroxybutyrate or poly(3-hydroxybutyrate)
PHBHHx	copolymers of 3-hydroxybutyrate and 3-hydroxyhexanoate
PHO	poly(3-hydroxyoctanoate)
PHBV or PHBHV	poly(3-hydroxybutyrate-*co*-3-hydroxyvalerate) or poly(hydroxybutyrate-*co*-hydroxyvalerate) or poly(3-hydroxybutyric acid-*co*-3-hydroxyvaleric acid)
3HV	3-hydroxyvalerate
MW	molecular weight
ZnO	zinc oxide
IPNs	interpenetrating polymer networks
PLA	polylactic acid
PTAcEt	poly(3-thiophene ethyl acetate)
PE	polyethylene
PHEMA	poly(hydroxyethyl methacrylate)
HEMA	hydroxyethyl methacrylate
GO	graphene oxide
rGO	reduced graphene oxide
CNTs	carbon nanotubes
SWCNTs	single-wall carbon nanotubes
MWCNTs	multi-wall carbon nanotubes
PHBV-g-MWCNTs	PHBV-grafted multi-walled carbon nanotubes
CNFs	carbon nanofibers
GNS	graphene nanosheets
CNCs	cellulose nanocrystals
CNFs	cellulose nanofibrils
NFC	nanofibrillated cellulose
POM	polarised optical microscope
PBAT	poly(butylene adipate-*co*-terephthalate)
RWF	recycled wood fibre
NC	nanoclay
WS_2_	tungsten disulphide
INT-WS_2_	tungsten disulphide inorganic nanotubes
BN	boron nitride
ABOw	aluminium borate whiskers
TiO_2_	titanium dioxide
SEM	scanning electron microscopy
TEM	transmission electron microscopy
PCL	poly(ε-caprolactone)
PBS	poly(butylene succinate)
TEC	triethyl citrate
DCP	dicumyl peroxide
UV	ultraviolet
CuO	copper oxide
CS	chitosan
COS	chitooligosaccharide
HAc	hyaluronic acid
SPIONs	superparamagnetic iron oxide nanoparticles
CEF	antibiotic ceftiofur
PEO	polyethylene oxide
Hap	hydroxyapatite
SCPL	solvent casting particulate leaching
SLS	selective laser sintering
FDM	fused deposition modelling
CAD	computer aided design
RP	rapid prototyping
µ-CT	microcomputerised tomography
CS	calcium silicate
